# Relationship between chronic pathologies of the supraspinatus tendon and the long head of the biceps tendon: systematic review

**DOI:** 10.1186/1471-2474-15-377

**Published:** 2014-11-18

**Authors:** Lucía Redondo-Alonso, Gema Chamorro-Moriana, José Jesús Jiménez-Rejano, Patricio López-Tarrida, Carmen Ridao-Fernández

**Affiliations:** Research group “Area of Physiotherapy CTS-305”, Department of Physiotherapy, University of Seville, C/ Rotonda de Santa Eufemia, n 35, Tomares, Seville Spain

**Keywords:** Tendinopathy, Supraspinatus, Long head of the biceps tendon, Chronic, Assessment

## Abstract

**Background:**

Chronic supraspinatus tendinopathy is a common clinical problem that causes functional and labor disabilities in the population. It is the most frequent cause of shoulder pain. This pathology may be frequently associated to the affectation of the long head of biceps tendon (LHBT), the main stabilizer of the glenohumeral joint together with the supraspinatus. The main aim of this work is to study the prevalence of lesions in LHBT associated to the chronic pathology of the supraspinatus tendon.

**Methods:**

A systematic review was carried out between May to July 2013 in the electronic databases: CINAHL, WOK, Medline, Scopus, PEDro, IME (CSIC) and Dialnet. The keywords used were: 1) in English: chronic, supraspinatus “long head of the biceps tendon”, biceps, rotator cuff, tendinosis, tendinopathy, evaluation, examination; 2) in Spanish: supraespinoso, biceps, tendinopatía. Inclusion criteria of the articles included subjects with a previously diagnosed chronic pathology of rotator cuff (RC) without previous surgery or any other pathologies of the shoulder complex. The total number of articles included in the study were five.

**Results:**

The results show an epidemiological relationship between both tendons. The age of the subjects included in the review was between 35 and 80 years, and some of the studies seem to indicate that the tendinopathy is more frequent in men than in women. The sample size of the studies varies according to the design, the highest being composed of 229 subjects, and the minimum of 28. Not all the articles selected specify the diagnostic testing, though the ones most normally used are arthroscopy, ultrasound, magnetic resonance imaging and assessment tests. The percentage of associated lesions of LHBT and supraspinatus tendon is between 78.5% and 22%, with a major prevalence in the studies with a smaller sample.

**Conclusions:**

The review of literature corroborates an association between the chronic pathology of the supraspinatus tendon and LHBT due to the epidemiological data. In addition, some authors confirm the existence of an anatomical and functional relationship between LHBT and the supraspinatus tendon, the latter being part of the LHBT pulley.

**Electronic supplementary material:**

The online version of this article (doi:10.1186/1471-2474-15-377) contains supplementary material, which is available to authorized users.

## Background

The supraspinatus muscle belongs to the rotator cuff muscles as well as the infraspinatus muscles, subscapularis, teres minor, and the long head of biceps tendon, although some authors do not include the latter[1–3]. It participates in shoulder abduction besides being involved in the compression of the humeral head against the glenoid as well as being considered one of the main stabilizers of the glenohumeral joint[1–5].

The prevalence of lesions in the rotator cuff (hereafter RC) is variable, increasing with age. Some epidemiological studies report an incidence of 5% in patients in their fourth decade and 80% in their eighth, with a predominance of chronic lesions[3]. Amongst all the RC pathologies, supraspinatus tendinopathies are the most common, appearing with a frequency of 61.9% in men and 38.1% in women[6–9].

Supraspinatus tendinopathies are the most common cause of shoulder pain, mainly causing pain and functional deficit, in people over 35 years old. Whatever the etiology of tendinopathy (impingement, micro-traumatism, vascularization, or degeneration), pain is the most relevant symptom[7, 8, 10].

The role of the Supraspinatus is of such importance, that when there is an injury or weakness thereof, the normal balance of forces acting on the glenohumeral joint is interrupted, thus causing instability to said joint. Thus, it triggers an instability in the long head of the biceps tendon, the main stabilizer of the glenohumeral joint, along with the supraspinatus[10–12].

On the other hand, the pathologies of the long head of the biceps tendon (LHBT) also present an important source of shoulder pain, often causing heavy losses in this joint flexion. LHBT tendinopathy is generally due to inflammatory, traumatic and degenerative causes related to overuse, becoming chronic in most cases[2].

Although LHBT ruptures represent 96% of all RC ruptures they rarely appear as isolated lesions and are often linked to other pathologies, such as supraspinatus tendinosis[13, 14]. Certain studies[15, 16] have found a close relationship between RC tears and associated injuries produced in LHBT. Bearing in mind that rotator cuff tears are thought to occur in up to 50% of the population, tendinopathy of the long head of biceps is considered a common clinical problem[17].

Besides the functional and structural problems caused by the pain, the high health cost caused by these lesions has to be taken into account, above all because they are long-lasting pathologies which tend to become chronic[13]. Furthermore, LHBT tendinosis presents very similar clinical symptoms to those of the supraspinatus, so that a correct differential diagnosis is important to achieve efficient treatment[14, 16, 18].

Therefore, a systematic review was performed with the main objective of studying the prevalence of LHBT lesions associated to the chronic pathology of the supraspinatus tendon. On the other hand, the secondary objective is to find studies in literature which indicate the existence of an anatomical and functional relationship between both tendons which would explain the association of both pathologies. All this will lead the clinical professionals to become aware of the associated lesions of both tendons, thus improving clinical practice, differential diagnoses as well as treatments for shoulder tendinopathies.

## Methods

A review of literature was performed in search of a prevalence of lesions associated to LHBT and the supraspinatus tendon. This was carried out by two blind reviewers, independent in respect to the other, with a degree of agreement of 100% regarding the articles.

The risk of bias of each study was assessed with the STROBE Statement[19] (Strengthening the Reporting of Observational Studies in Epidemiology) (Table 1).

**Table 1:** **STROBE statement (Strengthening the Reporting of Observational Studies in Epidemiology)**

	Murthi [16]	Singajaru [20]	Chelli [21]	Braun [22]	Modi [23]
**Title and abstract**	Yes	Yes	Yes	Yes	Yes
**Introduction**					
Background/rationale	Yes	Yes	Yes	Yes	Yes
Objectives	Yes	Yes	Yes	Yes	Yes
**Methods**					
Study design	Yes	Yes	Yes	Yes	Yes
Setting	Yes	No	Yes	Yes	No
Participants	Yes	Yes	Yes	Yes	Yes
Variables	Yes	Yes	Yes	Yes	Yes
Data sources	Yes	Yes	Yes	Yes	Yes
Bias	No	No	No	Yes	No
Study size	No	No	No	No	No
Quantitative variables	Yes	Yes	Yes	Yes	Yes
Statistical methods	Yes	Yes	Yes	Yes	Yes
**Results**					
Participants	Yes	Yes	Yes	Yes	Yes
Descriptive data	Yes	Yes	Yes	Yes	Yes
Outcome data	Yes	Yes	Yes	Yes	Yes
Main results	Yes	Yes	Yes	Yes	Yes
Others analyses	Yes	Yes	Yes	Yes	Yes
**Discussion**					
Key results	Yes	Yes	Yes	Yes	Yes
Limitations	No	Yes	Yes	Yes	Yes
Interpretation	Yes	Yes	Yes	Yes	Yes
Generalizability	Yes	Yes	Yes	Yes	Yes
**Other information**					
Funding	No	No	No	No	No
TOTAL	18/22	18/22	19/22	20/22	17/22

Risk of bias of the studies included in the review.

Finally, the methodological quality of this systematic review was assessed with the PRISMA checklist[24] (Table 2).

**Table 2:** **PRISMA checklist**

Section/topic	#	Checklist item	Page #
**TITLE**			
Title	1	Identify the report as a systematic review, meta-analysis, or both.	1
**Abstract**			
Structured summary	2	Provide a structured summary including, as applicable: background; objectives; data sources; study eligibility criteria, participants, and interventions; study appraisal and synthesis methods; results; limitations; conclusions and implications of key findings; systematic review registration number.	2
**Introduction**			
Rationale	3	Describe the rationale for the review in the context of what is already known.	3
Objectives	4	Provide an explicit statement of questions being addressed with reference to participants, interventions, comparisons, outcomes, and study design (PICOS).	3,4
**Methods**			
Protocol and registration	5	Indicate if a review protocol exists, if and where it can be accessed (e.g., Web address), and, if available, provide registration information including registration number.	-
Eligibility criteria	6	Specify study characteristics (e.g., PICOS, length of follow-up) and report characteristics (e.g., years considered, language, publication status) used as criteria for eligibility, giving rationale.	4
Information sources	7	Describe all information sources (e.g., databases with dates of coverage, contact with study authors to identify additional studies) in the search and date last searched.	4 + Table 1
Search	8	Present full electronic search strategy for at least one database, including any limits used, such that it could be repeated.	4 + Figure 1
Study selection	9	State the process for selecting studies (i.e., screening, eligibility, included in systematic review, and, if applicable, included in the meta-analysis).	4-5 + Figure 1
Data collection process	10	Describe method of data extraction from reports (e.g., piloted forms, independently, in duplicate) and any processes for obtaining and confirming data from investigators.	4 + Table 3
Data items	11	List and define all variables for which data were sought (e.g., PICOS, funding sources) and any assumptions and simplifications made.	4 + Table 2
Risk of bias in individual studies	12	Describe methods used for assessing risk of bias of individual studies (including specification of whether this was done at the study or outcome level), and how this information is to be used in any data synthesis.	4 + Table 3
Summary measures	13	State the principal summary measures (e.g., risk ratio, difference in means).	-
Synthesis of results	14	Describe the methods of handling data and combining results of studies, if done, including measures of consistency (e.g., I^2^) for each meta-analysis.	Table 3
Section/topic	#	Checklist item	Reported on page #
Risk of bias across studies	15	Specify any assessment of risk of bias that may affect the cumulative evidence (e.g., publication bias, selective reporting within studies).	4 + Table 3
Additional analyses	16	Describe methods of additional analyses (e.g., sensitivity or subgroup analyses, meta-regression), if done, indicating which were pre-specified.	-
**RESULTS**			
Study selection	17	Give numbers of studies screened, assessed for eligibility, and included in the review, with reasons for exclusions at each stage, ideally with a flow diagram.	5 + Figure 1
Study characteristics	18	For each study, present characteristics for which data were extracted (e.g., study size, PICOS, follow-up period) and provide the citations.	5,6 + Table 2
Risk of bias within studies	19	Present data on risk of bias of each study and, if available, any outcome level assessment (see item 12).	Table 3
Results of individual studies	20	For all outcomes considered (benefits or harms), present, for each study: (a) simple summary data for each intervention group (b) effect estimates and confidence intervals, ideally with a forest plot.	5,6,7 + Table 2
Synthesis of results	21	Present the main results of the review. If meta-analyses are done, include for each, confidence intervals and measures of consistency.	-
Risk of bias across studies	22	Present results of any assessment of risk of bias across studies (see Item 15).	Table 3
Additional analysis	23	Give results of additional analyses, if done (e.g., sensitivity or subgroup analyses, meta-regression [see Item 16]).	-
**Discussion**			
Summary of evidence	24	Summarize the main findings including the strength of evidence for each main outcome; consider their relevance to key groups (e.g., healthcare providers, users, and policy makers).	7,8
Limitations	25	Discuss limitations at study and outcome level (e.g., risk of bias), and at review-level (e.g., incomplete retrieval of identified research, reporting bias).	8
Conclusions	26	Provide a general interpretation of the results in the context of other evidence, and implications for future research.	8
**Funding**			
Funding	27	Describe sources of funding for the systematic review and other support (e.g., supply of data); role of funders for the systematic review.	-

### Inclusion criteria

Inclusion criteria of the selected articles were as follows:

- Studies carried out with humans.
- Subjects with chronic supraspinatus tendon or LHBT pathologies, both diagnosed medically.
- Subjects over 35 years old.
- Studies with a larger sample than 20 subjects.
- Patients with no previous surgery except arthroscopies for diagnosis purposes.
- Studies whose original language is English or Spanish.

### Exclusion criteria

The only exclusion criterion in the study is as follows:

→ Case studies.

The search for articles in databases was not restricted to the year of publication.

### Search strategy

An electronic search was performed for the months of May to July 2013 in the following databases: CINAHL, MEDLINE, Scopus, PEDro, WOK, IME (CSIC), Dialnet. Search terms and Boolean operators used are shown in Table 3.

**Table 3:** **Terms used and search strategies in the electronic databases**

Databases	MeSH* terms.	Search strategies
MEDline	1. Chronic	#2 AND #4 AND #7
WOK	2. Supraspinatus	#2 AND #3
CINAHL	3. “Long Head of the Biceps Tendon”	#1 AND #2 AND #4 AND (#6 OR #7)
Scopus	4. Biceps	#2 AND #4 AND (#8 OR #9)
PEDro	5. Rotator cuff	#4 AND #5 AND (#6 OR #7)
	6. Tendinosis	
	7. Tendinopathy	
	8. Evaluation	
	9. Examination	
	DeCS*^1^terms.	
CSIC (IME)	10. Supraespinoso	#10 AND #11
Dialnet	11. Bíceps	#10 AND #11 AND #12
	12. Tendinopatía	

*MeSH: Medical Subjects Headings.

* ^*1*^ *DeCS: Descriptores en Ciencias de la Salud.*

## Results

### Search results

**Figure 1:**
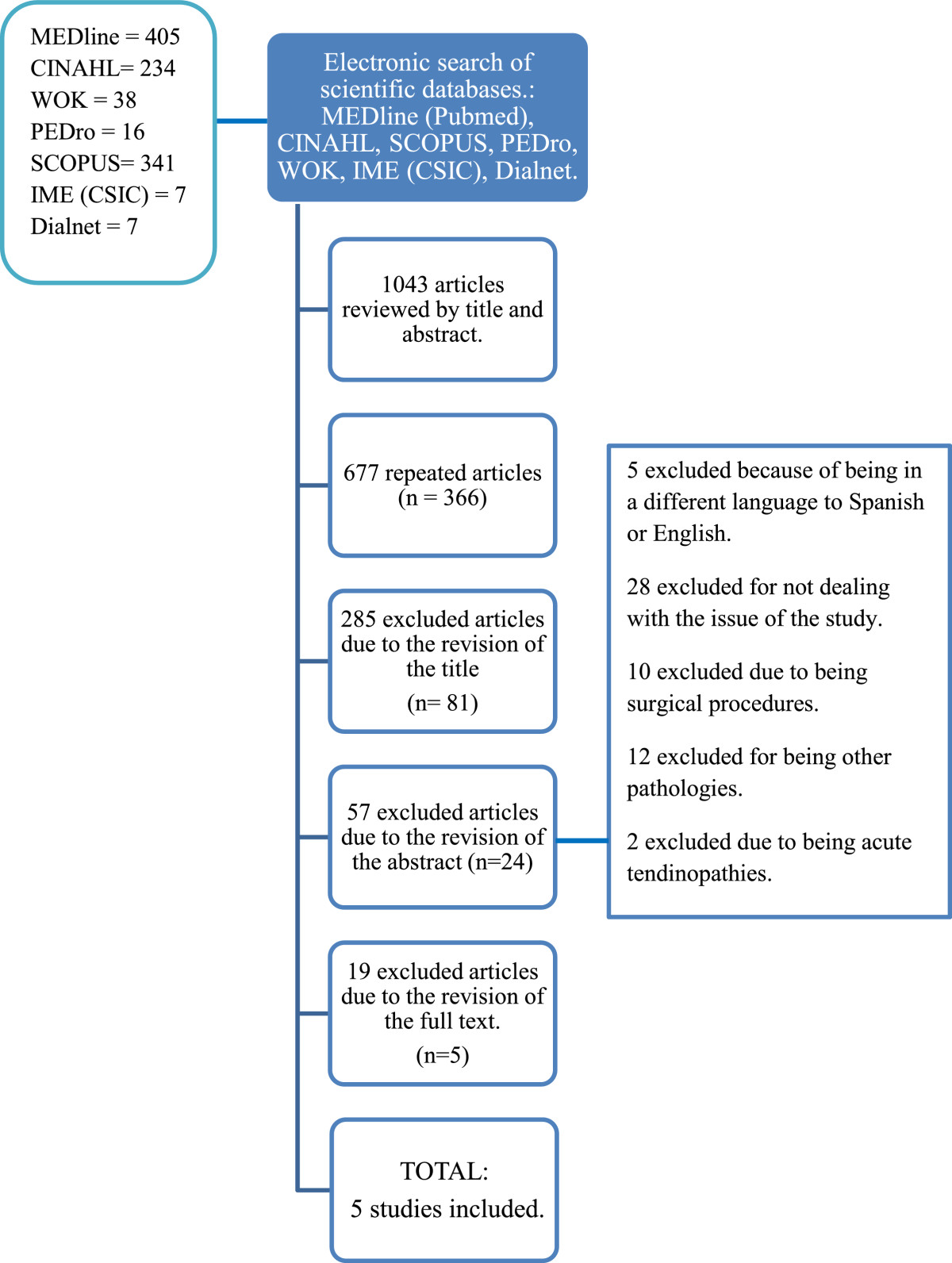
**Search strategy and selection of relevant articles.**

The initial search in electronic databases provided a total of 1043 items. Out of all the articles selected, 677 were repeated, so that 366 manuscripts remained to be analyzed. Next, those whose titles were unrelated to the topic were eliminated, leaving 81 works. A review of the summary in these articles was carried out, leaving 24 texts. Finally, 19 articles were ruled out after the full text review did not meet the inclusion criteria. The 5 studies finally included in the review were: Singajaru VM et al.[20], Murthi AM et al.[16], Modi CS et al.[23], Chelli BM et al.[21] and Braun S et al.[22]. The outline of the search strategy is shown in Figure 1.

### Characteristics of subjects

The age of the subjects included in the review was between 35 and 80 years, ages at which most chronic rotator cuff injuries occur[7, 8, 10].

Regarding sexes, in the study of Chelli BM et al.[23] there were 35 males and 29 females, and in the Braun S et al.[22] study more men were observed (n=155) than women (n=74). In Singajaru VM et al.[20], Murthi AM et al.[16] and Modi CS et al.[23] studies, this information is not specified.

The sample size varies considerably depending on the type of study, those of Murthi AM et al.[16] (n = 200), Modi CS et al.[23] (n = 100) and Braun S et al.[22] (n = 207) were very high; while it was lower in Chelli BM et al.[21] (n = 64) and Singajaru VM et al.[20] (n = 28).

Regarding the characteristics of the lesions that the subjects in each study present, we found that most participants had a clinical diagnosis of rotator cuff pathology. Once the muscles belonging to the rotator cuff were studied by means of invasive diagnoses (arthroscopy) and non-invasive (evaluation test, radiography, ultrasonography and magnetic resonance), we observed that the high percentage of involvement of the supraspinatus tendon and LHBT existed in the majority. In studies with fewer participants such as the one of Singajaru VM et al.[20], an association of both pathologies was observed in 78.5% of the subjects studied. However, the authors who evaluated a larger sample such as Braun S et al.[22] and Modi CS et al.[23] a lower percentage of lesions associated to both tendons (67.2% and 22%) was seen.

### Diagnostic tests used

All subjects from the articles analyzed were suffering from a chronic RC pathology, or LHBT lesion; both of which had been previously diagnosed by a physician[16, 20–23]. The 5 studies mention the diagnosis method. The most common are:

- The specific rating tests (Yegarson test, Speed test, Neer test and Hawkins test) used by Singajaru VM et al. [20] and Modi CS et al. [23] to evaluate the functional limitation.
- Magnetic resonance imaging, mentioned by Modi CS et al. [23] and Chelli BM et al. [21].
- The ultrasound used by Modi CS et al. [23] and Chelli BM et al. [21].
- Arthroscopy which is the most common process used [16, 20, 23].

Among the diagnosis methods used in assessing the shoulder, we found imaging diagnosis, especially by ultrasonography, corresponded to the gold standard[18].

### Variables

Regarding the variables analyzed in the selected 5 studies, all the authors focused on the description of morphological and histological changes of the tendons involved through imaging or arthroscopy, mainly concentrating their studies on the existence of tears, tendinopathies or inflammatory processes[16, 21–23]. Singajaru VM et al.[20], besides examining the above, also studied pain and the functional limitation.

### Synthesis of results

Murthi AM et al.[16], included an impingement syndrome and RC tendinopathy in their sample subjects, after previously undergoing arthroscopy. These subjects were divided into two groups: tenosynovectomy and tenodesis. In group 1 (n = 120), undergoing tenosynovectomy, 34% of the subjects had a partial RC tear, while 57% had a complete one. The 49% of all patients had associated LHBT with degenerative and inflammatory signs. In group 2 (n = 80), intervened by tenodesis, there were 63% with microscopic inflammatory changes, 9% of cases with signs of inflammation and calcification and 15% with fibrosis.

Singaraju VM et al.[20] determined the histological changes produced at LHBT level in persons diagnosed for RC tendinitis in their study, focusing especially on the supraspinatus tendon. To do this, they divided the 28 subjects into two equal groups, with the objective of comparing the subjects with RC affectation to those who did not. They found histological changes produced at LHBT level in a RC tendinitis in their study. Obviously microscopic inflammation changes were found, which ensured tendon damage, such as the presence of two neuropeptides involved in pain perception, CGRP and substance P, at the level of nerve endings in the inflamed tissues. Of 14 patients with supraspinatus affectation, 11 had damaged LHBT, finding a relationship between both lesions. According to the author, the difference between both groups was statistically significant (P = 0.04).

Next, the study performed by Chelli BM et al.[21] in 2010 similarly supported an association in the prevalence of both pathologies. Out of 64 subjects included in the study, 55 showed a supraspinatus pathology and among these, 16 showed alterations in LHBT.

Similarly, Braun S et al.[22] describes the pathology associated with LHBT affecting the biceps pulley, which the supraspinatus tendon forms part of and found 67 patients with biceps lesions and 45 with associated supraspinatus damage.

Finally, Modi CS et al.[23] analyzes the changes in all the RC tendons, including LHBT in subjects with previous shoulder arthroscopy. Out of 100 patients, 62 had supraspinatus tendon pathologies, of which 22 had a LHBT associated injury. This would imply an epidemiological relationship between both lesions that were found. Table 4 summarizes the characteristics and results of the studies included in our review.

**Table 4:** **Relevant characteristics of the studies included in the review**

Author and year	Design and duration	Subjects’ characteristics	Sample size	Diag ST/RC	Diag LHBT	Diagnostic test ST/RC	Diagnostic test LHBT	Variables	Results
*Murthi AM et al.*[16]	Incidence study, 4 years	Subjects with previous arthroscopy of subacromial syndrome or RC tendinopathy	200	Yes	No	Specific tests	Intra-articular LHBT arthroscopy	LHBT inflammation or degeneration	*Group 1:*
		Group 1: Arthroscopy with tenosynovectomy (mean age 47)				Physical findings			34% of subjects with partial rupture of the RC.
		Group 2: Arthroscopy with tenodesis (Mean age 55)				Arthrography		Pathology of RC	57% patients with complete rupture of the RC.
		Subject’s gender is not specified							49% patients with associated degenerative and inflammatory signs of LHBT
									*Group2:*
									63% of cases show microscopic changes of inflammation.
									9% cases with signs of inflammation and calcification.
									15% of the patients with fibrosis
*Singajaru, VM et al.*[20]	Histological study of cases and controls	*Intervention group:*	28	Yes	No		VAS scale	Shoulder pain	Presence of GCRP and Substance P (evidence inflammation)
		14 subjects with previous arthroscopy of the shoulder					Yegarson’s test	Functional limitation	The tendon and the sheath of 11 out of 14 subjects were affected (78.5%)
		Mean age 51-52					Speed’s test	Histological changes of the biceps tendon and sheath	
		*Control group:*					O’brien’s test		
		14 cadavers without RC alterations					Crank’s test		
		Mean age 72-76							
		Subject’s gender is not specified							
*Chelli BM et al.*[21]	Descriptive prevalence study 2 years	Subjects with previously diagnosed pathology of RC.	64	Yes		Ultrasonography MR	Ultrasonography MR	Most frequent age of lesion	55 patients with affectation of the ST (85.9%)
		Mean age 56 (35–75)						Most injured shoulder	16 patients with associated affectation of LHBT (29.1%)
		35 males and 29 females						Full and partial rupture of: supraspinatus tendon, infraspinatus tendon, subscapularis tendon and LHBT	
*Braun S. et al.*[22]	Cohort study	Subjects with shoulder pathologies	207	No	Yes	Arthroscopy	No	Pulley tears	67 patients were affected by biceps pulley (32.3%), of which 45 had alterations of the supraspinatus tendon (22%).
		Mean age 48,5 155 males and 74 females						Pulley tears Pulley width	
								Position of the biceps tendon	
								Other tendon injuries	
								RC injury	
*Modi CS et al.*[23]	Analytical retrospective	Patients with RC pathology following arthroscopy.	100	Yes		Hawkins’ test	Arthroscopy	Age	62 patients with thinning/lossof the supraspinatus tendon (62%)
		Without physiotherapy treatment for over 6 months.				Neer’s test		Kind of previous repair	22 lesions of LHBT (22%)
		Impingement signs +				Ultrasonography		Associated synovitis	
		Subjects over 35 years				MR		Biceps degeneration	
		Subject’s gender is not specified						Labrum degeneration	
								Other RC lesions	
								Osteoarthritis	

## Discussion

The selected studies were reviewed in order to determine the prevalence of LHBT lesions associated to the chronic pathology of the supraspinatus tendon.

First, we have found a very large number of articles as the topic in question was very specific and the search strategy as well as the choice of articles was a complex task. Currently, there are very few studies that relate both structures directly. However, the studies that we have included in this review have a similar design, as all of them are observational and cross descriptive studies. Thus, their methodological quality was assessed by the STROBE Statement[19] that is specific for this type of studies.

A large number of authors[11, 15, 16, 20] confirm the relationship between the supraspinatus tendon and LHBT, mainly the anatomical correlation. This is clear, as they are two essential structures in the stabilization of the humeral head in the glenoid. Furthermore, the supraspinatus tendon plays a basic role in stabilizing the LHBT, forming part of the reflection pulley itself[22, 25]. Although numerous studies[11, 15, 16, 20] confirm said anatomical relationship, only a few have decided to investigate it, which is why there are few conclusive results.

Regarding the overall objective of this review, the selected studies show a prevalence associated to the pathologies of each tendon. All of them included individuals with previous RC injuries, which include the supraspinatus muscle. Said investigations have focused on the assessment of the morphological changes of these structures, while always finding LHBT alterations in the form of tears, tendinopathies or subluxations amongst them[16, 18, 20–23].

According to the reviewed studies, the prevalence of associated lesions of LHBT and supraspinatus tendon varies considerably. On the one hand, Braun S et al.[23] show a percentage of associated damage between LHBT and supraspinatus tendon of 22%, being the larger sample study (n = 207). On the other hand, the research of Singajaru VM et al.[20], with a smaller sample (n = 28), presents a higher percentage of associated injuries than the former (78.5%).

The percentage of associated lesions may be influenced not only by the number of study subjects, but also by the characteristics of the sample, such as age or the diagnostic test used which may determine the associated prevalence of both pathologies.

According to the literature reviewed, older subjects are more predisposed to having associated lesions of both structures due to their chronicity, thus impacting on the overall biomechanics of the shoulder joint injury[20, 24]. On the other hand, those patients who have been diagnosed by arthroscopy will have a greater risk of involving both tendons as it is an invasive method. In addition, this can influence the performance of the shoulder stabilizing muscles[16, 18].

In spite of the disadvantages of the arthroscopy, we observe that it is the most common diagnostic method in shoulder pathologies. However, the percentage of double lesion of supraspinatus and LHBT does not seem to be related to the form of diagnosis in the studies included in the review. The imaging diagnosis is used in a second place, and it presents a good resolution of shoulder structures without disadvantages.

Murthi AM et al.[16] states that the incidence of chronic LHBT pathology in the painful shoulder is high, especially in the intra-articular portion, which is easily diagnosed by arthroscopy. Thus, LHBT should be considered in the assessment of patients with chronic RC pathologies. This idea is supported by Ji JH et al.[18], who believes that biceps tendinopathy is not taken into account when performing differential diagnosis of “shoulder pain”. Without a correct diagnosis, therefore, the treatment of shoulder injuries is not efficient.

Meanwhile, Singajaru VM et al.[20] alleges that a LHBT alteration is the leading cause of anterior shoulder pain. This is accompanied by damage to other structures surrounding it, such as the sheath and other RC tendons, specially the supraspinatus.

On the other hand, Modi CS et al.[23] showed in their study that supraspinatus tendinopathy is related to the subjects’ advanced age, with the eldest being most affected. Furthermore, LHBT degeneration is associated with bursa injuries. This statement is corroborated by Chelli BM et al.[21].

Braun S et al.[22] studied the relationship between LHBT and the reflection pulley of the latter which is essential for LHBT stability. They found a significant correlation between LHBT lesions and damage to the pulley and the supraspinatus tendon, since it forms part of it. Therefore, this author affirms the anatomical and functional relationship of said structures.

Regarding the diagnostic procedures, some items do not clearly specify what means were used[20–22]. Magnetic resonance imaging and arthroscopy, useful for tendon repair, were the most frequently mentioned[16, 18, 21–23].

Finally, this review presents a number of limitations. In regard to the search in literature, 5 articles had to be excluded for being written in a different language to English or Spanish (Italian, Turkish, German); nevertheless, not being part of the final phase but of the summary review, the final selection was not significantly affected. Regarding the results, few studies show a direct relationship between both structures, although an epidemiological relationship of both pathologies is shown, which could be due to the anatomical correlation confirmed by some authors. Furthermore, quantative measuring of data could not be performed as the characteristics of the studies included did not allow it, thus we have carried out a qualitative synthesis of the results obtained.

## Conclusions

A review of the selected literature seems to support an association between the chronic pathology of the supraspinatus tendon and LHBT, which is shown through the epidemiological data. These data could be crucial in clinical practice, implying an improvement in the differential diagnosis of both structures, which present very similar symptoms, and therefore, improve treatments.

Certain authors confirm the existence of an anatomical relationship between LHBT and the supraspinatus tendon, the latter forming part of the LHBT reflection pulley. Furthermore it could also be a functional relationship as both tendons are involved in the stabilization of the humeral head, and the damage to either one could affect the function of the other.

Given the prevalence associated to the pathologies of both tendons, we believe it is of great clinical interest to study prospectively the chronology of these lesions and their order of appearance, so as to prevent double lesions.

## Authors’ information

Research group “Area of Physiotherapy CTS-305”. Department of Physiotherapy. University of Seville (Seville – Spain).

## Authors’ original submitted files for images

Below are the links to the authors’ original submitted files for images. Authors’ original file for figure 1

## Abbreviations

LHBT: Long Head of the Biceps Tendon
RC: Rotator Cuff
ST: Supraspinatus tendon
MR: Magnetic resonance
AP: Anteroposterior
WOK: Web of Knowledge
PED: Physiotherapy Evidence Database
SMI: Spanish Medical Institute.
