# Supplementary material for: Relationship between chronic pathologies of the supraspinatus tendon and the long head of the biceps tendon: systematic review
Source: BMC Musculoskelet Disord. 2014 Nov 18;15:377. doi: 10.1186/1471-2474-15-377 (PMC4247626; doi:10.1186/1471-2474-15-377)
Supplement: Supplementary file 1 — Authors’ original file for figure 1 [file 12891_2013_2323_MOESM1_ESM.pdf]

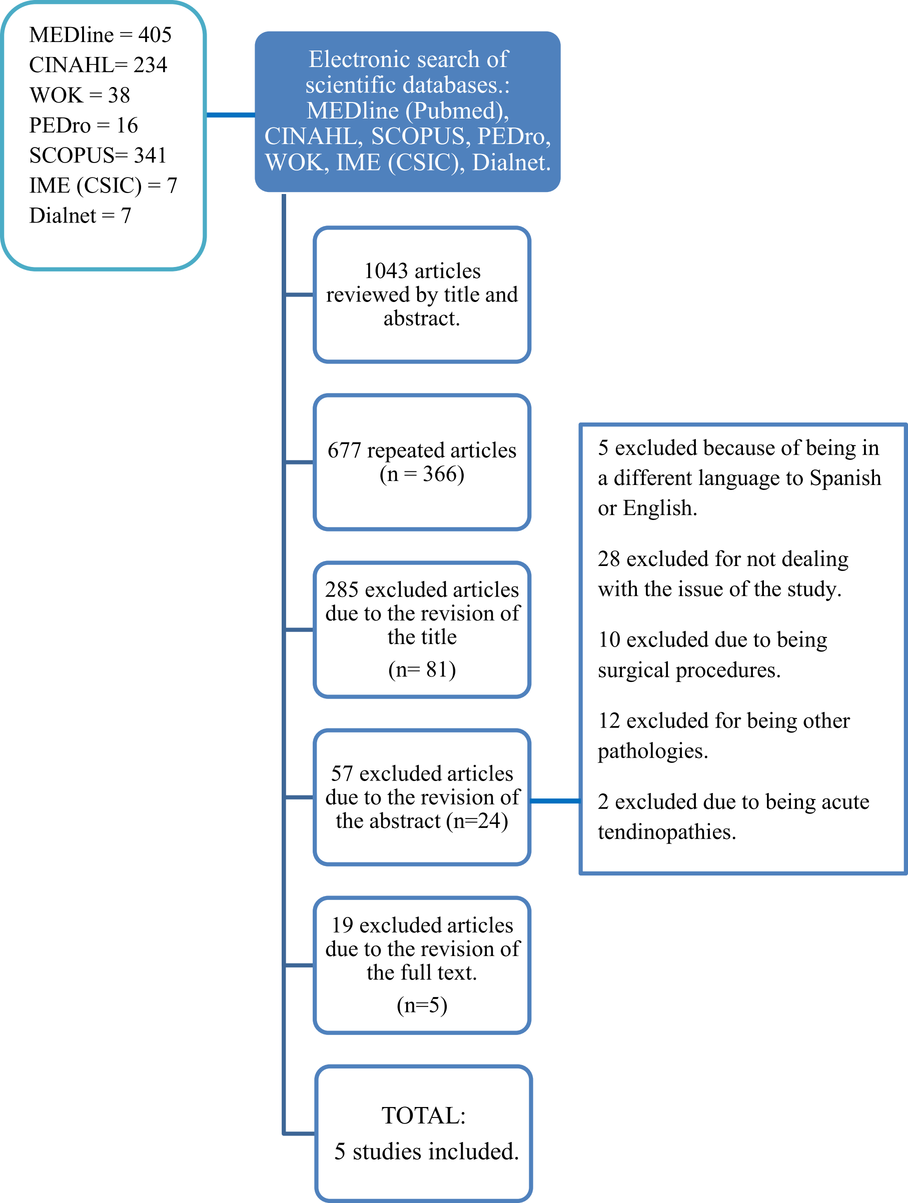
MEDline = 405
CINAHL= 234
WOK = 38
PEDro = 16
SCOPUS= 341
IME (CSIC) = 7
Dialnet = 7
Electronic search of scientific databases.: MEDline (Pubmed), CINAHL, SCOPUS, PEDro, WOK, IME (CSIC), Dialnet.
1043 articles reviewed by title and abstract.
677 repeated articles (n = 366)
285 excluded articles due to the revision of the title (n= 81)
57 excluded articles due to the revision of the abstract (n=24)
19 excluded articles due to the revision of the full text. (n=5)
TOTAL: 5 studies included.
5 excluded because of being in a different language to Spanish or English.
28 excluded for not dealing with the issue of the study.
10 excluded due to being surgical procedures.
12 excluded for being other pathologies.
2 excluded due to being acute tendinopathies.
